# The Views of Hospital Secretaries

**Published:** 1911-06-17

**Authors:** 


### THE VIEWS OF HOSPITAL SECRETARIES.

**Mr. FRED. NEDEN, Secretary and Manager of the County Hospital, York.**

The approval with which the National Insurance Bill appears to have been received by all political parties in the House of Commons is significant of the fact that, as a whole, the principles of the Bill are acceptable to the majority. But the promoters appear to have altogether lost sight of the immense amount of work the voluntary hospitals of the country have accomplished, and are still doing, in the alleviation of the evils the Bill is intended to remedy, and except for some very meagre provisions giving authority to approved friendly societies to grant subscriptions to hospitals, their existence appears to have been ignored.

It is well known to hospital managers that the subscriber who uses his " notes " obtains value far in excess of the amount of his contribution, and it may reasonably be expected that full advantage will be taken of the privileges given in respect of the donations, etc., which friendly societies may feel disposed to make.

According to paragraph 4 of clause 12, it is permissible for the sick benefit to which an insured person is entitled to be handed over by the Health Committee to the authorities of the convalescent home or sanatorium in which he may be a patient. Even if this arrangement were made applicable to general hospitals the amount of the benefit (which I conclude will be ten shillings per week) would be about one-third the average cost of in-patients. It seems fairly evident, therefore, that as the Bill now stands, the support hospitals may expect to receive from either of the two sources just referred to would be in the nature of an inadequate *quid pro quo* for benefits conferred.

Where is the balance to come from? This is a question which will cause great anxiety to hospital committees, and however undesirable it may be to many of us, the question of rate aid or State aid will be a factor to be considered. If either becomes an accomplished fact I fear the spirit of charity will be quenched, and one of the finest monuments to Britain's greatness—the voluntary care of the sick—will gradually disappear.

It is difficult to say what effect the Bill will have upon the Workpeople's Hospital Fund subscriptions, but I have great hopes that as far as this hospital is concerned the finances will not suffer materially, though it is impossible to see that any benefit will ensue. The York Workpeople's Hospital Fund is managed by a separate Committee and is so excellently organised that I believe the members will not permit the Fund to languish.

